# Efficacy of add-on mepolizumab in adolescents with severe eosinophilic asthma

**DOI:** 10.1186/s13223-019-0366-x

**Published:** 2019-09-03

**Authors:** Steven W. Yancey, Hector G. Ortega, Oliver N. Keene, Eric S. Bradford

**Affiliations:** 1 Respiratory Therapeutic Area, GSK, 5 Moore Drive, PO Box 13398, Research Triangle Park, NC 27709 USA; 2 Respiratory US Medical Affairs, GSK, La Jolla, CA USA; 3 Clinical Statistics, GSK, Stockley Park, Middlesex UK; 4 Present Address: GossamerBio, San Diego, CA USA

**Keywords:** Adolescent, Asthma control, Eosinophils, Exacerbations, Mepolizumab, Severe eosinophilic asthma

## Abstract

Adolescents (12–17 years of age) with severe eosinophilic asthma experience frequent exacerbations and reduced lung function leading to poor health-related quality of life. Mepolizumab is approved for add-on maintenance therapy in patients with severe eosinophilic asthma ≥ 6 years of age in the EU and ≥ 12 years of age in other regions (including the USA), based on a Phase II/III program demonstrating reduced exacerbation rates with 4-weekly treatment. A total of 34 adolescent patients were recruited across the Phase III mepolizumab trials. Consistent with outcomes in the overall population, there was a reduction in the annual rate of clinically significant exacerbations, along with a reduction in blood eosinophil counts in response to mepolizumab in adolescent patients. The safety profile in adolescent patients was consistent with that seen in the overall population. Data from the Phase III clinical development program provide evidence for comparable efficacy and safety of mepolizumab between adolescents with severe eosinophilic asthma and the overall population.

*Clinical trial registration* DREAM, NCT01000506 [MEA112997]; MENSA, NCT01691521 [MEA115588]; SIRIUS, NCT01691508 [MEA115575]; MUSCA, NCT02281318 [200862]; COSMOS, NCT01842607 [MEA115661].

Dear editor,

Asthma is a substantial health problem among adolescents (12–17 years of age) [1]. Despite receiving maximum standard of care therapy, adolescents with severe asthma and eosinophilic inflammation experience poor symptom control, reduced lung function and frequent exacerbations, often leading to compromised health-related quality of life [2–4]. In particular, frequent exacerbations are disabling for the patient and their caregivers/family, typically requiring treatment with high doses of oral corticosteroids (OCS) and, in many cases, hospital admission [5, 6]. Regular use of OCS (> 7 mg/day) is associated with a number of well-documented side effects, one of which is growth impairment, a particular concern for adolescents [7]. Although the prevalence of adolescents within the severe eosinophilic asthma population is low (approximately 4% when based on recruitment to clinical trials) [8, 9], the disease burden for this sub-population is substantial [6]. Evidence is provided by the observation of more frequent hospitalizations required for adolescents with severe asthma than for adult patients with severe asthma [5, 6]. Furthermore asthma also has a significant impact in adolescents due to days lost at school [10]. Poor self-care is an important contributory factor, as well as treatment compliance [11–13].

Several biologic therapies are now available for patients with severe asthma who were previously without effective treatment options. Mepolizumab, an anti-interleukin (IL)-5 therapy, was approved in 2015 for add-on maintenance treatment of patients with severe asthma ≥ 12 years of age and with an eosinophilic phenotype; in 2018 the EU license was extended to include patients with severe asthma ≥ 6 years of age. The Phase II/III program for mepolizumab showed that mepolizumab administered once every 4 weeks reduced exacerbation rates and OCS use, and improved quality of life, compared with placebo in patients ≥ 12 years of age with severe eosinophilic asthma [14–17]. Here we present data from the adolescent population enrolled in this Phase II/III program.

Due to the low prevalence of severe eosinophilic asthma in adolescents, recruitment of adolescents into the Phase II/III mepolizumab trials was challenging. However, a total of 34 adolescent patients ≥ 12–17 years of age were randomized across 4 studies: the 32-week MENSA study (n = 25; NCT01691521); the 24-week MUSCA study (n = 9; NCT02281318); the 52-week DREAM study (n = 1; NCT01000506); and the 24-week SIRIUS study (n = 2; NCT01691508). In these trials, every 4 weeks, patients with severe eosinophilic asthma received either placebo or 75, 250 or 750 mg intravenous (IV) mepolizumab (DREAM); placebo or 75 mg IV mepolizumab or 100 mg subcutaneous (SC) mepolizumab (MENSA); or placebo or 100 mg mepolizumab (SIRIUS/MUSCA). Full details of the inclusion/exclusion criteria and treatment schedules have been previously published [14–17].

Adolescents in the MENSA study (the study with the largest recruitment of adolescents) displayed similar baseline exacerbation rates, Asthma Control Questionnaire (ACQ) scores and blood eosinophil counts compared with the overall population. However, in the adolescent population, asthma duration was shorter (as expected), a numerically higher proportion of patients were atopic and experienced hospitalizations associated with exacerbations, and a numerically lower proportion of patients were using maintenance OCS at baseline (Table 1).

**Table 1:** Baseline demographics and disease characteristics for patients in MENSA and in DREAM/MENSA/MUSCA/SIRIUS combined

	MENSA			DREAM/MENSA/MUSCA/SIRIUS		
	12–17 years (N = 25)	≥ 18 years (N = 551)	Overall population (N = 576)	12–17 years (N = 37)	≥ 18 years (N = 1841)	Overall population (N = 1878)
Age, years, mean (SD)	15 (2)	52 (12)	50 (14)	15 (2)	51 (12)	50 (13)
Asthma duration, years, mean (SD)	9.6 (4.2)	20.4 (13.9)	19.9 (13.8)	10.3 (3.9)	19.6 (14.3)	19.5 (14.2)
Atopic^a^, n (%)	18 (72)	252 (46)	270 (47)	28 (76)	853 (46)	881 (47)
Exacerbations in year prior to study, mean (SD)	3.7 (2.8)	3.6 (2.6)	3.6 (2.6)	3.7 (2.5)	3.3 (2.6)	3.3 (2.6)
Patients experiencing an exacerbation requiring ED visit/hospitalization, n (%)	10 (40)	180 (33)	190 (33)	21 (57)	653 (35)	674 (36)
Patients experiencing an exacerbation requiring hospitalization, n (%)	8 (32)	101 (18)	109 (19)	13 (35)	406 (22)	419 (22)
ACQ score at baseline, mean (SD)	1.9 (1.1)	2.2 (1.2)	2.2 (1.2)	1.9 (1.2)	2.3 (1.2)	2.3 (1.2)
Maintenance OCS use at baseline, n (%)	4 (16)	140 (25)	144 (25)	6 (16)	592 (32)	598 (32)
Baseline blood eosinophil count, cells/μL, geometric mean (SD logs)	240 (0.91)	300 (1.00)	290 (0.99)	290 (0.90)	280 (1.03)	280 (1.03)

^a^Atopy defined as testing ≥ 0.35 kU/L for house dust mite, dog dander, cat dander, or *Alternaria alternate* allergens

ACQ: Asthma control questionnaire; ED: emergency department; OCS: oral corticosteroids; SD: standard deviation

Eosinophilia has been defined as a key phenotypic characteristic of patients with severe eosinophilic asthma [16, 18]. While the proportion of patients with atopy was numerically different between the adolescent group and the overall population, the blood eosinophil count appeared similar. The Phase III mepolizumab program demonstrated selective inhibition of eosinophilic inflammation and reduction in the number of eosinophils in both sputum and blood in patients treated with mepolizumab [14, 16, 17]. A reduction in blood eosinophil counts in response to mepolizumab treatment was also demonstrated in the adolescent population in MENSA from Week 4; the count reached 40 cells/μL at Week 32, which was consistent with the overall population. Furthermore, a population pharmacokinetic (PK) analysis of MENSA data indicated no effect of age on the PK of mepolizumab, with adolescents displaying plasma concentrations consistent with adults and predicted clearance within the range of the rest of the study population, irrespective of administration route (data not shown).

Blood eosinophil count was also shown to be a predictive biomarker for response to mepolizumab treatment, with reductions in eosinophil counts accompanied by reductions in clinically significant exacerbations [14, 16, 17]. In a post hoc subgroup analysis of the two studies that recruited adolescent patients in each randomized treatment group (MENSA and MUSCA), the adolescent population showed generally similar reductions in the annual rate of clinically significant exacerbations compared with patients ≥ 18 years of age (Fig. 1). In a post hoc meta-analysis of patients from the MENSA and MUSCA trials, overall there was a 40% reduction in the annual rate of clinically significant exacerbations in the adolescent population, 12–17 years of age, consistent with the reduction shown in adult patients, ≥ 18 years of age (Fig. 1). As these studies were not designed to detect differences between mepolizumab and placebo in the adolescent population, no formal tests of statistical significance were performed.

**Fig. 1:**
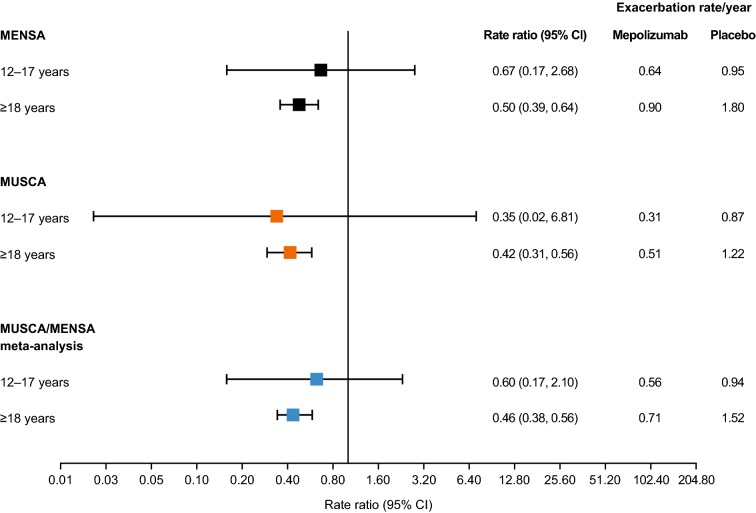
Clinically significant exacerbation rate/year in adolescent and adult patients in the MENSA and MUSCA trials. Analyses were conducted using a negative binomial regression model including covariates for use of maintenance oral corticosteroids, geographic region, number of exacerbations in the previous year, baseline percentage of the predicted FEV_1_ (MENSA and MUSCA) and treatment (MENSA only). Increases in 95% CIs observed in adolescent groups are a result of the smaller sample size when compared with ≥ 18 years; CI: confidence interval; FEV_1_: forced expiratory volume in 1 s

In terms of safety, over the course of the Phase III program, mepolizumab was shown to be well tolerated in the overall population [14–17], and the safety profile was similar in the adolescent population. The incidence and profile of adverse events (AEs) and serious AEs experienced by adolescents (n = 37) was similar to those reported in the overall populations in the primary studies (MENSA, MUSCA, DREAM, SIRIUS) [14–17]. There were no withdrawals due to AEs among adolescents receiving placebo or mepolizumab, and the most common AEs in this group were headache, nasopharyngitis, and upper abdominal pain. Five adolescent patients receiving placebo or mepolizumab reported serious AEs (asthma exacerbation [n = 3], gastroenteritis rotavirus [n = 1], dyshidrotic eczema [n = 1]); none of these AEs were considered to be treatment-related, and of these serious AEs, only one case of asthma exacerbation and one of dyshidrotic eczema was reported in patients receiving mepolizumab. Of the 37 adolescent patients enrolled in the primary studies, 26 entered COSMOS (NCT01842607) [19], a 52-week, open-label extension study of MENSA and SIRIUS in which all patients received SC mepolizumab 100 mg, and have long-term safety data available. Adolescent patients had a median of 12.1 months of exposure to mepolizumab 100 mg SC, ranging from 2 to 20 months. In COSMOS, the AE and serious AE profile for adolescents remained similar to the overall population, with nasopharyngitis, sinusitis, and asthma the most commonly reported AEs. Five adolescent patients experienced serious AEs, the most commonly reported of which were asthma exacerbations (n = 5/26; 19%). This is a numerically higher percentage than that observed in the overall COSMOS population for the serious AE of asthma exacerbation (n = 38/651; 6%) [19]; however, this observation should be considered with caution as the adolescent sub-group was small.

Given the substantial unmet treatment need in adolescent patients with severe eosinophilic asthma, it is important to determine how biological therapies that have demonstrated efficacy in adult patients with severe eosinophilic asthma perform in the adolescent population. Although the adolescent population was small, data from the Phase III mepolizumab clinical development program provide evidence for comparable efficacy and safety of mepolizumab between adolescents with severe eosinophilic asthma and the overall population.

## Data Availability

Anonymized individual participant data and study documents for the original studies can be requested for further research from http://www.clinicalstudydatarequest.com.

## Abbreviations

ACQ: Asthma Control Questionnaire
AE: adverse event
CI: confidence interval
ED: emergency department
FEV_1_: forced expiratory volume in 1 s
IL-5: interleukin-5
OCS: oral corticosteroids
PK: pharmacokinetic
SC: subcutaneous
SD: standard deviation
